# A Multiplex Assay for the Stratification of Patients with Primary Central Nervous System Lymphoma Using Targeted Mass Spectrometry

**DOI:** 10.3390/cancers12071732

**Published:** 2020-06-29

**Authors:** Daniel M. Waldera-Lupa, Gereon Poschmann, Nina Kirchgaessler, Omid Etemad-Parishanzadeh, Falk Baberg, Mareike Brocksieper, Sabine Seidel, Thomas Kowalski, Anna Brunn, Aiden Haghikia, Ralf Gold, Anja Stefanski, Martina Deckert, Uwe Schlegel, Kai Stühler

**Affiliations:** 1 Institute of Molecular Medicine, Medical Faculty, Heinrich-Heine-University, 40225 Düsseldorf, Germany; daniel.waldera@hhu.de (D.M.W.-L.); gereon.poschmann@hhu.de (G.P.); Nina.Kirchgaessler@hhu.de (N.K.); Omid.Etemad-Parishanzadeh@hhu.de (O.E.-P.); f.baberg@web.de (F.B.); Mareike.Brocksieper@hhu.de (M.B.); 2 Department of Neurology, Knappschaftskrankenhaus, Ruhr-University Bochum, 44789 Bochum, Germany; Sabine.Seidel@kk-bochum.de (S.S.); thomas.kowalski@kk-bochum.de (T.K.); uwe.schlegel@kk-bochum.de (U.S.); 3 Institute of Neuropathology, Faculty of Medicine and University Hospital Cologne, University of Cologne, 50937 Cologne, Germany; anna.brunn@uni-koeln.de (A.B.); neuropatho@uni-koeln.de (M.D.); 4 Department of Neurology, St. Josef-Hospital, Ruhr-University Bochum, 44789 Bochum, Germany; aiden.haghikia@rub.de (A.H.); r.gold@klinikum-bochum.de (R.G.); 5 Molecular Proteomics Laboratory, Biologisch-Medizinisches Forschungszentrum, Heinrich-Heine-University Düsseldorf, 40225 Düsseldorf, Germany; Anja.Stefanski@hhu.de

**Keywords:** primary central nervous system lymphomas, secondary central nervous system lymphomas, multiple sclerosis, glioma, cerebrospinal fluid, biomarker, diagnosis, proteomics

## Abstract

Primary central nervous system lymphomas (PCNSL) account for approximately 2% to 3% of all primary brain tumors. Until now, neuropathological tumor tissue analysis, most frequently gained by stereotactic biopsy, is still the diagnostic gold standard. Here, we rigorously analyzed two independent patient cohorts comprising the clinical entities PCNSL (*n* = 47), secondary central nervous system lymphomas (SCNSL; *n* = 13), multiple sclerosis (MS, *n* = 23), glioma (*n* = 10), other tumors (*n* = 17) and tumor-free controls (*n* = 21) by proteomic approaches. In total, we identified more than 1220 proteins in the cerebrospinal fluid (CSF) and validated eight candidate biomarkers by a peptide-centric approach in an independent patient cohort (*n* = 63). Thus, we obtained excellent diagnostic accuracy for the stratification between PCNSL, MS and glioma patients as well as tumor-free controls for three peptides originating from the three proteins VSIG4, GPNMB4 and APOC2. The combination of all three biomarker candidates resulted in diagnostic accuracy with an area under the curve (AUC) of 0.901 (PCNSL vs. MS), AUC of 0.953 (PCNSL vs. glioma) and AUC 0.850 (PCNSL vs. tumor-free control). In summary, the determination of VSIG4, GPNMB4 and APOC2 in CSF as novel biomarkers for supporting the diagnosis of PCNSL is suggested.

## 1. Introduction

Primary central nervous system lymphomas (PCNSL) account for approximately 2% to 3% of all primary brain tumors [1] and mainly affect elderly patients with a rising incidence in patients older than 60 years [2]. PCNSL are extranodal lymphomas and more than 90% represent highly malignant non-Hodgkin’s lymphoma of the diffuse large B-cell (DLBCL) type [3]. PCNSL carry a less favorable prognosis than systemic DLBCL and are characterized by possible dissemination within the brain, spinal cord, leptomeninges and cerebrospinal fluid (CSF); infiltration of the vitreous and chorioretina affects about 10% of patients and is considered as a manifestation of central nervous system (CNS) lymphoma [4].

In the last three decades, the prognosis of PCNSL patients has improved substantially due to the development and application of methotrexate-based chemotherapy regimens [5]. Accurate diagnosis is essential for treatment planning. At times, radiological differential diagnosis of PCNSL from inflammatory diseases such as "tumefactive" multiple sclerosis (MS) lesions may be equally as challenging as discrimination from other tumors [1,6]. Neuropathological and molecular tumor tissue analysis from biopsy material is the diagnostic gold standard, which bears the risk of severe injuries and infections. Classification of PCNSL includes the immunohistochemical demonstration of B-cell markers such as CD19, CD20, CD79a, a late GC exit phenotype (CL6, MUM1), expression of BCL2, Myc and a high proliferative activity [7]. There is a growing body of experimental data from global as well as targeted approaches suggesting alternative molecular biomarkers for in vitro diagnostics of PCNSL. The analysis of putative biomarkers in serum (IL-6, IL-8, IL-10, CXCL-12 and CXCL 13) and CSF (IL-10, CXCL-12 and CXCL 13) revealed aberrant protein levels in association with PCNSL [8,9,10,11,12]. Furthermore, the occurrence of ectodomains from transmembrane proteins such as CD27 and sIL-2R were discovered in the CSF of PCNSL patients, which may represent candidate biomarkers [9,11,13,14,15].

Here, we present a combined discovery and validation approach for the development of a diagnostic assay using CSF—a biomaterial that is acquired with lower potential risks than biopsies—allowing discrimination of PCNSL patients from other clinical entities with high accuracy using a mass spectrometry-based proteomic approach.

## 2. Results

For the generation of an in vitro diagnostic assay allowing the stratification of PCNSL patients, we rigorously analyzed five clinical groups diagnosed with PCNSL, secondary CNS lymphoma (SCNSL), multiple sclerosis (MS), glioma or other tumors as well as tumor-free individuals by an integrated discovery and validation approach using quantitative mass spectrometry (Table 1a and Table S1A). First, we determined the individual proteome signature of the CSF from all patients (*n* = 67) by a shotgun proteomics approach and in the second step, we selected candidate biomarkers which were validated in an independent cohort (*n* = 64; Table 1b and Table S1B) by targeted mass spectrometry using selected reaction monitoring (SRM) and immunohistochemistry.

### 2.1. Analysis of BBB Dysfunction in the Discovery Cohort

**Figure 1:**
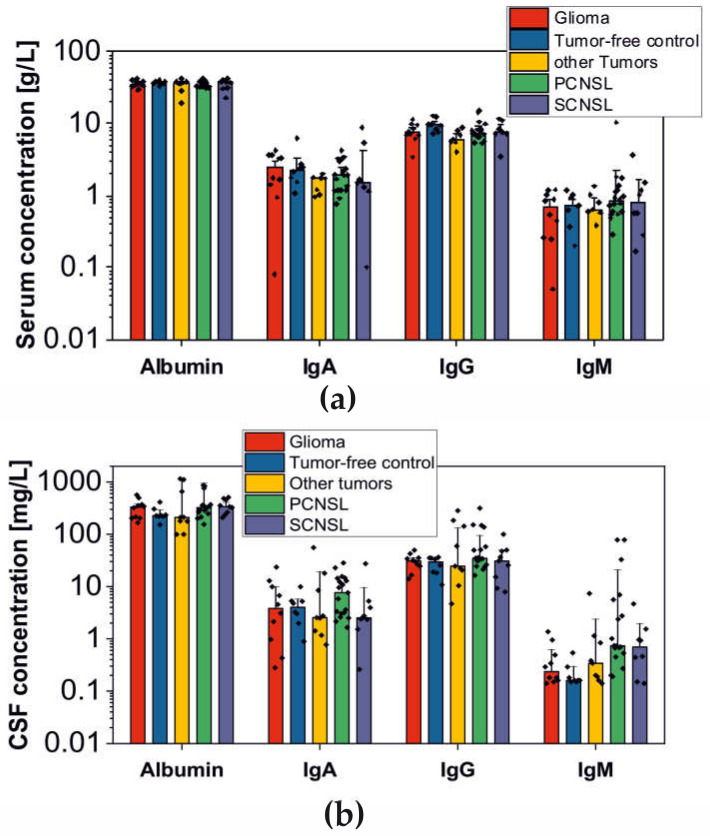
Overview of protein concentration obtained by ELISA (discovery cohort). The data was considered for the determination of blood–brain barrier (BBB) dysfunction. Median values and standard errors are shown (primary central nervous system lymphomas (PCNSL): *n* = 19, secondary central nervous system lymphomas (SCNSL): *n* = 9, glioma: *n* = 10, other tumors: *n* = 10, tumor-free control: *n* = 8). (**A**) Concentrations of albumin, IgG, IgA and IgM in serum and (**B**) cerebrospinal fluid (CSF).

Frequently, PCNSL and other brain tumors cause blood–brain barrier (BBB) dysfunction, which leads to plasma protein leakage into the CSF [13,16,17]. To reveal the extent of BBB dysfunction in individual patients, we determined the CSF/serum quotients of albumin, IgG, IgA and IgM concentration (Figure 1 and Table 2). In the discovery study cohort, albumin was significantly increased and immunoglobulins (IgG, IgA, IgM) were slightly elevated in PCNSL tumor patients in contrast to tumor-free control patients and confirmed that in 58% of the analyzed PCNSL patients, the BBB is disrupted. The proportion of patients exhibiting BBB dysfunction was similar in the glioma (60%) and SCNSL (56%) groups. In the group “other tumors", the proportion (40%) was slightly lower. In the control group (tumor-free), only one out of eight patients (13%) with idiopathic intracranial hypertension exhibited BBB dysfunction. For the MS patient group, we did not calculate CSF/serum quotients of albumin, IgG, IgA and IgM concentration as the CSF contains inherently more IgG [18]. This high degree of BBB dysfunction in the analyzed clinical entries underlined the necessity to consider leakage of plasma proteins into the CSF to avoid the selection of false-positive candidate biomarkers for further validation.

### 2.2. Discovery Study: Identification of Candidate Biomarkers by Quantitative Mass Spectrometry

Next, we established individual proteome signatures of the CSF from patients diagnosed with PCNSL (*n* = 19), SCNSL (*n* = 9), MS (*n* = 9), glioma (*n* = 9), other tumors (*n* = 10) and without tumor (*n* = 8) by a quantitative mass spectrometry-based proteomic approach. The “other tumor” group includes samples from patients with meningeosis carcinomatosa (mammary carcinoma), primitive neuroectodermal tumors, desmoplastic medulloblastoma and plasmocytoma (Table S1A). Using label-free mass spectrometric quantification, we identified 1220 proteins (10,437 peptides, Table S2) in the CSF, and we quantified 569 proteins (7317 peptides). As shown above, the BBB was disrupted in more than 50% of tumor samples, so we excluded all peptides correlating with CSF albumin as candidate plasma leakage proteins from the analysis. In total, 375 quantified proteins (2284 peptides) significantly (*p* ≤ 0.001) correlated with CSF albumin and were not considered further for biomarker validation. For candidate plasma leakage proteins (375 proteins), we confirmed that at least 86% originated from the four plasma-associated tissues (UniProt tissue annotation database) (Table S3).

**Figure 2:**
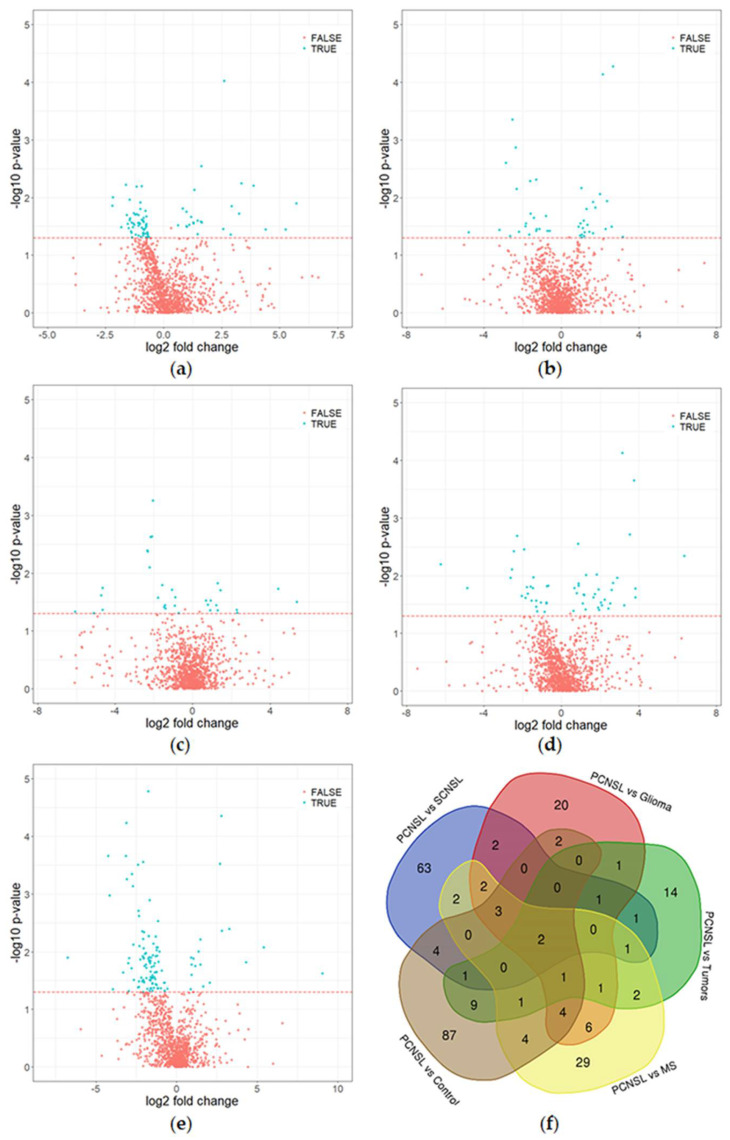
Volcano plots and Venn diagram of differential proteome analysis (discovery cohort). (**a–e**): The red line indicates a *p*-value of *p* = 0.05. Peptides marked as “true” (*p* < 0.05, turquoise) differed significantly in abundance between the PCNSL patients and respective groups, whereas proteins marked with “false” (red) exhibit no significant abundance change. (**a**) PCNSL vs. SCNSL, (**b**) PCNSL vs. glioma, (**c**) PCNSL vs. “other tumors”, (**d**) PCNSL vs. multiple sclerosis (MS), (**e**) PCNSL vs. non-tumor controls. (**f**) Venn diagram of significantly altered peptides.

Furthermore, we decided to follow a peptide-centric approach to establish a diagnostic assay for the differential diagnosis of PCNSL patients, as it offers the opportunity to select appropriate biomarker molecules from a much larger group of candidates (5033 peptides in comparison with 194 proteins) to establish a reliable validation assay. First, we performed a statistical analysis (ANOVA) of the complete data set to select the appropriate candidate peptide biomarkers and to exclude overlapping candidate biomarkers. This analysis revealed that 82 (PCNSL vs. SCNSL), 45 (PCNSL vs. Glioma), 35 (PCNSL vs. “other tumors”), 58 (PCNSL vs. MS) and 118 (PCNSL vs. control) peptides were significantly (*p* ≤ 0.05) altered between the analyzed patient groups (Table 3, Figure 2A–E). Overall, only two peptides (hemoglobin subunit delta (HBD) and amyloid-like protein 2 (APLP2)) were found to be differentially abundant in all patient groups (Figure 2F). Biological characterization of the candidate biomarkers confirmed the results of our previous study [13] that CNS proteins (64%) are significantly altered in the CSF of PCNSL patients in comparison with non-disease controls. This holds also true for the comparison of PCNSL with SCNSL (51%), PCNSL with gliomas (56%), PCNSL with “other tumors” (55%) and PCNSL with MS (52%) (Table 2). We also identified a high number of secreted and membrane proteins among the differentially abundant proteins which is in concordance with our former observations [13].

As the second criterion for the selection of candidate biomarkers, we considered the diagnostic accuracy determined by the ROC analysis of the individual peptide intensities determined by label-free mass spectrometry (Table 4, Figure S1A–E). Considering the area under the curve (AUC) being greater than 0.7, we determined 7 (PCNSL vs. SCNSL), 11 (PCNSL vs. glioma), 5 (PCNSL vs. “other tumors”), 7 (PCNSL vs. MS) and 5 (PCNSL vs. control) peptides as potential candidates for the differential diagnosis of the respective groups (Table 4, Figure S1A–E). The candidate biomarker peptides were mostly exclusive for the respective patient groups (Figure S1F). From these candidate biomarkers, we selected 33 candidate peptide biomarkers for further evaluation. As targeted mass spectrometric analysis offers high specificity for peptide identification and quantification in complex protein mixtures as well as direct transferability for candidate validation, we decided to consider selected reaction monitoring (SRM) analysis [19].

### 2.3. Validation of Candidate Peptide Biomarker in an Independent Patient Cohort Using SRM

**Figure 3:**
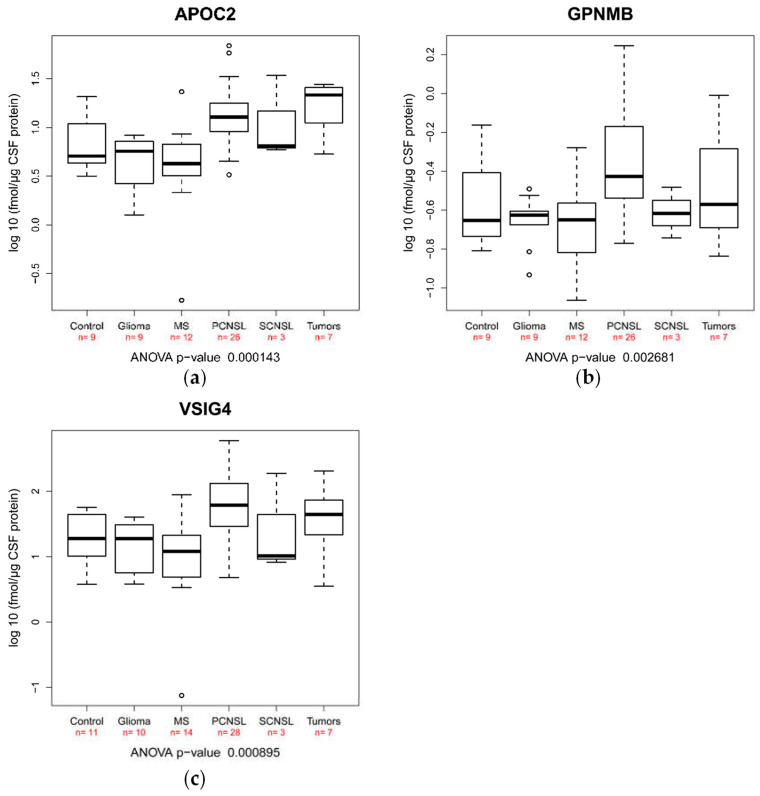
Boxplots of the candidate biomarker peptides’ concentration, using SRM-based absolute quantification on the concentration of candidate biomarker peptides: (**a**) APOC, (**b**) GPNMB and (**c**) VSIG4. Boxes include 50% of all values for the respective group and the black bar in the box represents the median value of log 10 concentration values. The end of the upper whiskers represents the smallest value of the maximum value and maximum upper quartile plus 1.5*inter quartile range (IQR) (min(max(x), Q_3 + 1.5 * IQR)); the end of the lower whisker represents the larger value of the minimum value and minimum lower quartile minus 1.5xinter quartile range (max(min(x), Q_1 – 1.5 * IQR)). The number of valid values for each group and peptide/protein is given.

To obtain reliable quantification results, we screened our 33 candidate peptide biomarkers regarding their performance for SRM analysis considering oxidizable amino acids, missed cleavage sites as well as theoretical transition interference. Finally, we selected 19 peptides as likely suited for SRM analysis (Table 4). As we intended an absolute quantification approach (AQUA) based on SRM analysis, we considered 19 corresponding heavily labeled peptides of our candidate biomarkers. During the establishment of the SRM assay, we excluded 11 additional peptides as it turned out those peptides either did not provide a reasonable signal-to-noise ratio for quantification (eight peptides) or suffered from larger transition interferences under our experimental conditions (three peptides). Finally, eight peptides exhibited a good performance in the quantitative assays with concentrations in the range from 0.35 to 67.8 fmol/µg CSF protein (Table 5). For the validation of the candidate biomarkers, we used an independent patient cohort of 63 samples (Table S1B). The “other tumors” samples included samples from patients with meningeosis carcinomatosa (mammary carcinoma), desmoplastic medulloblastoma and rectal cancer cerebral metastasis. We revealed that under the chosen experimental set-up, three different peptides confirmed significant differences found in the discovery study (AYVPIAQVK from GPNMB, ANOVA *p*-value 0.0026; GSDPVTIFLR from VSIG4, ANOVA *p*-value 0.00089 and TAAQNLYEK from APOC2, ANOVA *p*-value 0.00014; Table 6, Figure 3). By Tukey’s post hoc tests, we revealed that these three candidate biomarkers exhibit significant differences in the comparison between PCNSL vs. glioma as well as PCNSL vs. MS (Table 6). As mentioned above and confirmed by our patient cohorts, MS is more frequently diagnosed at a younger age as compared with PCNSL. Therefore, we included age as a variable in the variance analysis and did not find a significant influence on peptide concentrations in CSF for AYVPIAQVK from GPNMB, GSDPVTIFLR from VSIG4 and TAAQNLYEK from APOC2 (*p*-values 0.79, 0.94 and 0.44, respectively). Although we have a high drop-off rate for peptide markers due to the reasons mentioned above, we successfully validated three candidate biomarkers in an independent patient cohort.

### 2.4. Multiplexed SRM Assay for the Diagnosis of PCNSL Using CSF Samples

**Figure 4:**
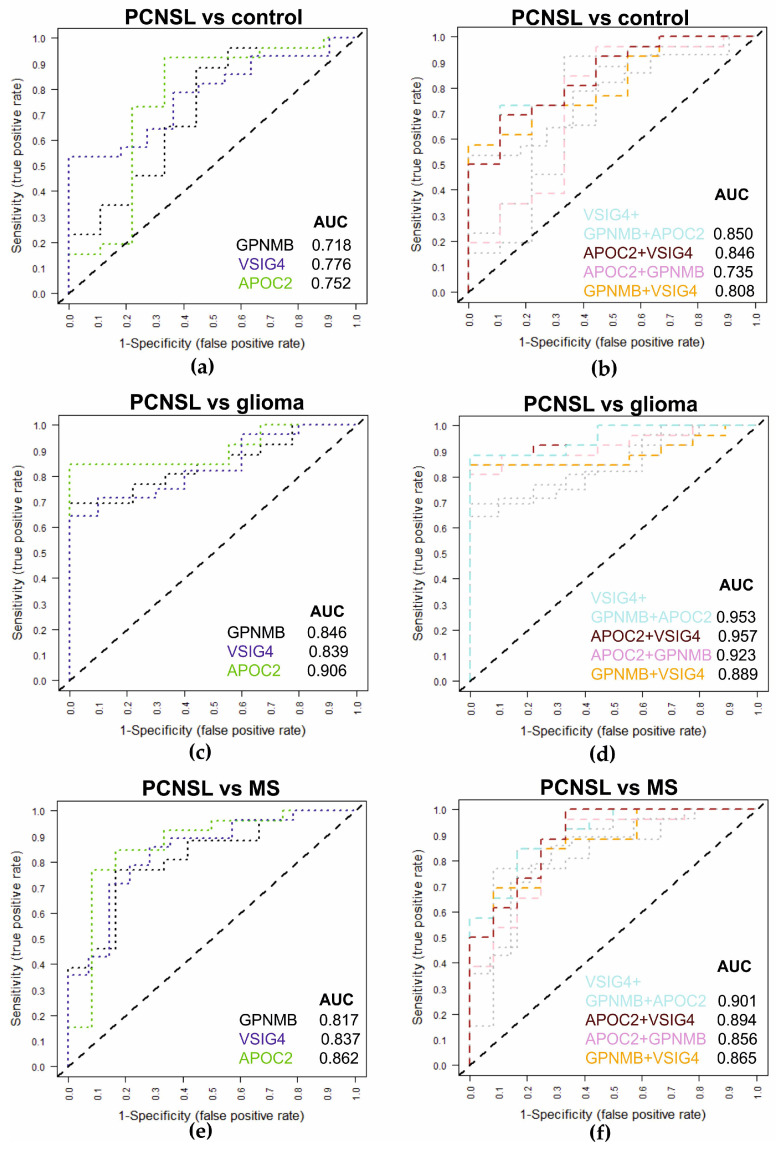
Diagnostic qualities of candidate peptide biomarkers determined by receiver operating characteristic (ROC) analysis. Comparison of peptide concentrations between samples from: (**a**,**b**) PCNSL and control patients, (**c**,**d**) PCNSL and (**e**,**f**) glioma patients and PCNSL and MS patients. Curves and area under the curve (AUC) values of single peptides (**a**,**c**,**e**) as well as curves of combinations of three or two markers (colored) as well as single peptides (grey, curves also in panel a) (**b**,**d**,**f**).

As the three candidate biomarkers revealed significant abundance changes in CSF between PCNSL and MS as well as glioma patients, respectively, we analyzed the candidate peptide biomarkers according to their diagnostic accuracy using our validation cohort sample set. ROC analysis of each marker peptide (Figure 4A,C,E) revealed that the individual marker peptides can discriminate PCNSL from the control patient samples (AUC: 0.718 GPNMB; 0.776 VSIG, 0.752 APOC). The discriminative power between PCNSL and glioma patients (AUC: 0.846 GPNMB; 0.839 VSIG, 0.906 APOC) as well as PCNSL and MS patients (AUC: 0.817 GPNMB; 0.837 VSIG, 0.862 APOC) was even better. Moreover, we determined the individual concentration cut-offs for the best diagnostic performance based on the data of the validation sample set as here absolute marker amounts were available. Based on the heavy labeled standard peptides comparing PCNSL with glioma patients, we obtained for AYVPIAQVK (GPNMB) a sensitivity of 69.2% and specificity of 88.8% by a cut-off of 0.0.32 fmol/µg. For the VSIG4 peptide GSDPVTIFLR, a sensitivity of 71.4% and specificity of 90.0% can be estimated at a cut-off of 36.2 fmol/µg protein and TAAQNLYEK (APOC2) can discriminate PCNSL from glioma patients at a cut-off of 8.3 fmol/µg with a sensitivity and specificity of 84.6% and 77.7%, respectively. For the comparison of PCNSL with MS patients, the sensitivities and specificities were 76.9%, and 83.3% (GPNMB, cut-off 0.29 fmol/µg), 78.6% and 78.6% (VSIG4, cut-off 27.5 fmol/µg) and 84.6% and 83.3% (APOC2, cut-off 8.3 fmol/µg). PCNSL patients could further be discriminated from non-tumor controls with sensitivities and specificities of 65.4% and 66.6% (GPNMB, cut-off 0.37 fmol/µg), 64.3% and 72.7% (VSIG4, cut-off 40,7 fmol/µg) and 73.1% and 77.8% (APOC2, cut-off 10.9 fmol/µg). Next, we tested if the combination of the candidate biomarkers can improve the discriminative power [20]. Thus, we demonstrated that the combination of two or all three candidate biomarkers improved the discrimination power in all cases (Figure 4B,D,E). For the comparison of PCNSL with the control patient samples, the combination of all three markers (APOC2/GPNMB/VSIG4) resulted in an AUC of 0.850 and the combination of VSIG1 and APOC2 in an AUC of 0.846. A significant improvement in discriminative power was reached for the comparison of PCNSL and glioma samples (APOC2/GPNMB/VSIG4: AUC 0.953, APOC2/VSIG: 0.957) and PCNSL and MS samples (APOC2/GPNMB/VSIG4: AUC 0.901, APOC2/VSIG: 0.894).

### 2.5. Immunohistochemistry of Candidate Biomarkers

**Figure 5:**
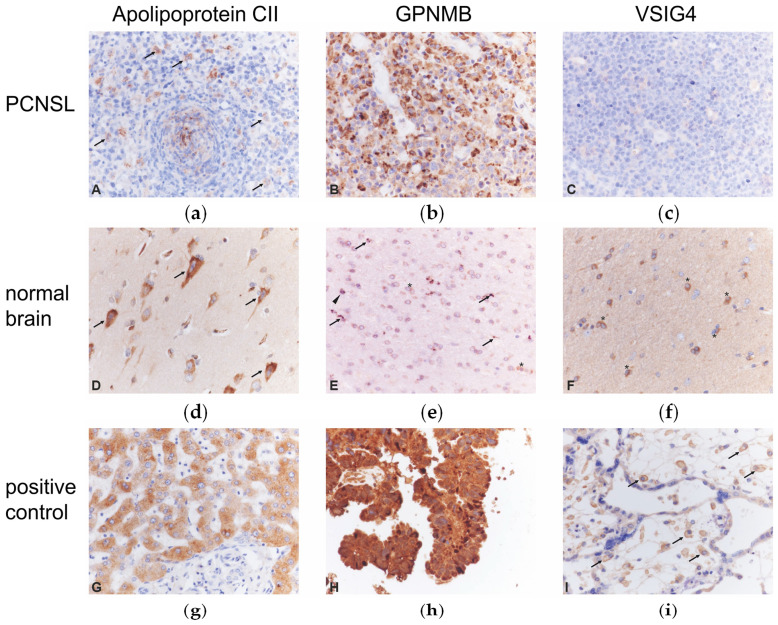
Protein distribution of apolipoprotein CII, GPNMB and VSIG4 in PCNSL and normal brain: (**a**) apolipoprotein CII in PCNSL, (**b**) GPNMB in PCNSL, (**c**) VSIG4 in PCNSL, (**d**) apolipoprotein CII in normal brain, (**e**) GPNMB in normal brain, (**f**) VSIG4 in normal brain, (**g**) apolipoprotein CII in positive control, (**h**) GPNMB in positive control, (**i**) VSIG4 in positive control. In PCNSL, apolipoprotein CII is expressed by a few tumor cells (**a, arrows**) and GPNMB by numerous tumor cells (**b**). The tumor cells do not express VSIG4 (**c**). In the normal brain, cortical neurons show a cytoplasmic expression of apolipoprotein CII (**d, arrows**). Microglial cells (arrows), an oligodendrocyte (arrowhead) and astrocytes (asterisks) express GPNMB (**e**). Astrocytes show a cytoplasmic expression of VSIG4 (**f, asterisks**). Normal hepatic tissue, colon carcinoma, and placenta were used as control tissue for apolipoprotein CII (**g**), GPNMB (**h**), VSIG4 (**i, arrows**), respectively. Immunohistochemistry with polyclonal rabbit anti-apolipoprotein CII (Abcam, Cambridge, UK), rabbit anti-GPNMB (clone E4D7P, Cell Signaling, Frankfurt, Germany), and polyclonal rabbit anti-VSIG4 (Novus Biologicals, Wiesbaden, Germany); slight counterstaining with hemalum; original magnification ×400.

To determine the cellular origin of the candidate proteins and validate our observation that most identified candidate proteins in our discovery study stemmed from the CNS, normal brain and PCNSL tissue specimens were analyzed by immunohistochemistry. As proof-of-principle, we investigated the expression pattern of apolipoprotein CII, GPNMB and VSIG4. In the normal brain, astrocytes expressed apolipoprotein CII, GPNMB and VSIG4 (Figure 5, Table 7). Further, GPNMB was expressed by microglia and neurons. Concerning PCNSL, 30% (6/20) showed tumor cell expression of GPNMB. Apolipoprotein CII was detected in 10% (2/20) PCNSL: in one of these cases, < 50% of the tumor cells were immunoreactive, while in the other case, only single tumor cells showed cytoplasmic immunoreactivity. All PCNSL of this series (100%, 20/20) were consistently negative for VSIG4.

Thus, these proof-of-principle studies demonstrate that resident brain cell populations, i.e., astrocytes, microglia and neurons, account for GPNMB, apolipoprotein CII and VSIG4 expression, whereas only GPNMB was expressed in a significant fraction of PCNSL by the tumor cells.

## 3. Discussion

In this study, we rigorously analyzed two independent patient cohorts by two orthogonal proteomic approaches for the discovery (*n* = 74 patients) and validation (*n* = 63) of candidate CSF peptide biomarkers as a diagnostic tool in PCNSL patients. By a shotgun proteomic approach, we established a CSF proteome from PCNLS patients with more than 1220 proteins which exceed a former study that had identified around 500 proteins [21]. Removal of 375 plasma proteins which likely appear as a result of plasma leakage due to BBB disruption allowed us to avoid false-positive candidate biomarkers [17]. Detailed data analysis and immunohistochemistry confirmed the results from a previous study which showed that more than half of the proteins with significant abundance changes in the CSF of PCNSL patients originated mainly from the surrounding CNS tissue instead of the lymphoma tissue [13]. Here, we speculate that the lymphoma tissue interacts and communicates with its environment (CNS tissue) that causes the release of proteinaceous factors by different secretory pathways (ectodomain shedding).

The combination of two orthogonal mass spectrometric platforms allowed us to transfer the results from the discovery study directly to the candidate biomarker validation. With the peptide-centric approach, we were able to consider 2284 peptides for downstream biomarker validation. Finally, the optimization of the SRM assay and validation in an independent patient cohort (*n* = 63) yielded three candidate biomarkers (GSDPVTIFLR -VSIG4, AYVPIAQVK-GPNMB, TAAQNLYEK -APOC2) which exhibit significant abundance changes between the analyzed patient groups. These peptides/proteins have not been linked to PCNSL biology before, but it is interesting to note that two of the selected candidates (VISG4 and GBNMB) were detected in the CSF of PCNSL patients, most likely due to ectodomain shedding [13]. GPNMB, also known as glycoprotein nonmetastatic melanoma protein B or hematopoietic growth factor inducible neurokinin-1 type is constitutively expressed in the brain [22], as confirmed by our immunohistochemistry experiments. Increased mRNA and protein levels of GPNMB in the biopsy samples of patients with glioblastoma multiforme correlated with a higher survival rate [23]. The selected tryptic peptide AYVPIAQVK (position 219–227) of the ectodomain from GPNMB confirmed the results from a previous study [13] as it is significantly more abundant in the CSF of PCNSL patients in comparison with MS and glioma patients and exhibited excellent performance in the SRM assay. From V-set and immunoglobulin domain-containing protein 4 (VSIG4), the tryptic peptide GSDPVTIFLR at position 61–70 was found highly abundant in the CSF of the PCNSL patients in comparison with MS patients. VSIG4 is a phagocytic receptor and a strong negative regulator of T-cell proliferation and IL2 production [24]. VSIG4 is abundant in reactive astrocytes and therefore we speculate whether the increased amount of VSIG4 in comparison with MS may reflect a different inflammatory state of these diseases. The tryptic peptide TAAQNLYEK from apolipoprotein C-II (APOC2) was found to be higher abundant in the CSF of the PCNSL patients in comparison with MS and glioma patients. The immunohistochemistry results confirmed our observation that the candidate biomarkers originating from the surrounding tissue as APOC2 staining was only detected in normal brain tissue. APOC2 plays an important role in lipoprotein metabolism and activates the lipoprotein lipase to hydrolyze triglycerides [25]. Elevated APOC2 levels have also been found in the CSF of progressive MS subtypes in comparison with relapsing subtypes and have been associated with enhanced inflammation and elevated markers like IL-2 and IL-16 and eotaxin-3/CCL26 [26].

Finally, we tested whether the combination of peptide biomarkers in a multiplex assay improves the discriminative power of the SRM assay as a single marker did not exhibit sufficient power to discriminate the analyzed patient groups. Although two of the peptide markers seem to be associated with PCNSL biology due to ectodomain shedding, we selected these biomarkers mainly due to their discriminative power. In a previous study aiming for the diagnosis of epithelial ovarian cancer, it has been shown that a panel of five candidate proteins (each protein quantified with between one and three peptides) resulted in an AUC of 0.869 [27]. Here, we demonstrate that the combination of the concentration of three biomarkers in a peptide-centric approach provided excellent diagnostic accuracy for the differentiation of clinically relevant entities with a sensitivity of 85% and a specificity of 83% (PCNSL vs. MS) and a sensitivity of 88% and a specificity of 89% (PCNSL vs. glioma), and showed that an SRM-based assay can distinguish PCNSL from other clinical entities.

## 4. Materials and Methods

### 4.1. Patients, Clinical Data and CSF Collection

All CSF samples were obtained from the Department of Neurology, Knappschaftskrankenhaus Bochum (Bochum, Germany), and the Department of Neurology, St. Josef-Hospital Bochum; Ruhr-University (Bochum, Germany). This project was granted by the Ethics Committee of the Ruhr-University Bochum and all patients gave written informed consent. CSF was collected by a standard operating procedure. Briefly, CSF was collected by a lumbar puncture at ambient room temperature, and the first 10 drops were discarded to avoid blood contamination. CSF was immediately centrifuged at 500× *g* at 4 °C for 10 min to precipitate cell debris. Afterward, supernatants were aliquoted and stored at 80 °C. The whole procedure was performed within 30 min. For the discovery study, CSF samples from 65 patients (10 tumor-free controls; 19 PCNSL, 9 SCNSL, 9 MS, 10 gliomas, 10 other tumors) were included (Table 1a) (for detailed information, see Table S1A). For the composition of disease groups, we aimed for age and gender matching. The concentrations of albumin, IgG, IgA and IgM in serum and CSF were determined by the Knappschaftskrankenhaus Bochum via turbidity measurement (Roche Cobas 6000/Tina-quant, Roche, Mannheim, Germany) or a Nephelometer BN II (Siemens Healthineers, Erlangen, Germany) according to the manufacturers’ instructions. For the validation study, CSF samples from additional 63 patients (28 PCNSL, 3 SCNSL, 7 other tumors, 14 MS, 10 glioma and 11 tumor-free controls) were included (Table 1b) (for detailed information, see Table S1B). In the validation study, we considered novel sample preparations from identical glioma patients as in the discovery cohort. For the immunohistochemistry study, CSF samples from additional 25 patients (20 PCNSL, 5 tumor-free controls) were included (Table 1c). The immunohistochemistry study was approved by the Ethics Committee of the University Hospital of Cologne (06-187, 07-109) and performed according to the Declaration of Helsinki.

### 4.2. Sample Preparation

The CSF samples were prepared as already reported [13]. Briefly, the protein concentration of the CSF samples was determined by a Pierce 660 nm Protein Assay as described in the manufacturer’s protocol (Thermo Fisher Scientific, Rockford, IL, USA). For protein digestion, each sample containing 20 µg of protein was diluted to 25 µL with 50 mM ammonium hydrogen carbonate and 25 µL of 100% 2,2,2-trifluoroethanol followed by a subsequent reduction by adding 0.3 µL 1.4 M dithiothreitol at 42 °C for 60 min. Afterward, the reduced samples were alkylated by adding 4.2 µL 55 mM iodoacetamide and incubation at room temperature for 30 min in the dark. Each sample was then diluted 10-fold with 50 mM ammonium hydrogen carbonate before proteolysis. The proteins were digested with trypsin (weight ratio trypsin: protein 1:50) at 37 °C for 4 h. Proteolysis was stopped by the addition of 0.5 µL 10% trifluoroacetic acid (TFA). The solvent was completely removed with a vacuum concentrator (Eppendorf Concentrator 5301, Eppendorf, Hamburg, Germany). Each sample was reconstituted in 20 µL 0.1% TFA before liquid chromatography coupled mass spectrometric analysis. For validation, synthetic heavy peptides were spiked into the digested samples at a concentration of 16 fmol/µg CSF protein. Before measurement, the samples were randomized in their injection order.

### 4.3. Liquid Chromatography Coupled Mass Spectrometric Analysis - Discovery

For each liquid chromatography coupled mass spectrometric run, 500 ng sample was analyzed with a nano-high-performance liquid chromatography (HPLC)/ESI-mass spectometry system composed of an RSLCnano U3000 HPLC and a QExactive Plus mass spectrometer (Thermo Fisher Scientific, Bremen, Germany) equipped with a nano-electrospray ion source. Each sample was loaded onto a trapping column (Acclaim PepMap C18, 2 cm × 100 μm × 3 μm particle size, 100 Å pore, Thermo Fisher Scientific, Bremen, Germany) and desalted with 0.1% TFA for 10 min. Peptides were eluted from the trapping column, separated by an analytical column (Acclaim PepMap RSLC C18; 25 cm × 75 μm × 2 μm particle size, 100 Å pore; Thermo Fisher Scientific, Bremen, Germany) at a constant flow rate of 300 nL/min for 120 minutes and sprayed into the mass spectrometer. The mobile phase for chromatography consisted of 0.1% formic acid in water and 84% acetonitrile and 0.1% formic acid in water. The parameters for QExactive plus were as follows: positive mode; mass range of 350–2000 m/z with a resolution of 70.000 (precursor) or 200–2000 m/z with a resolution of 17.500 (fragment); spray voltage, 1.4 kV; ion transfer tube temperature, 250 °C; collision gas, helium; collision gas pressure, 1.3 mTorr; normalized collision energy for fragmentation, 30%; and isolation of +2, +3, +4 monoisotopic precursors with a width of 2.0 Da. TOP10 data-dependent acquisition with activated dynamic exclusion (repeat count 1, duration 100 ms) was applied.

### 4.4. Identification and Quantification

For protein identification, Proteome Discoverer (version 1.4.1.14, Thermo Fisher Scientific, Bremen, Germany) and the MS Amanda search engine were considered. Fragment spectra were searched against the UniProtKB/Swiss-Prot database (human; including isoforms; date 16 February 2017, 42,095 entries). The following search parameters were applied: enzyme, trypsin (full); maximum missed cleavage sites, 2; precursor mass tolerance, 10 ppm; fragment mass tolerance, 10 ppm; oxidation of methionine and deamidation of asparagine and glutamine as dynamic modifications; carbamidomethyl at cysteine as a static modification. The false discovery rate was set to 5%. Label-free quantification of peptides was performed with Progenesis QI for Proteomics (Version 2.0, Nonlinear Dynamics, Waters Corporation, Newcastle upon Tyne, UK). The liquid chromatography coupled mass spectrometric runs were automatically aligned with the software. If necessary, the alignment was manually corrected. Liquid chromatography coupled mass spectrometric runs were normalized by the software based on the assumption that most of the peptides were unchanged between the patients. Quantitative data of peptides were further statistically analyzed.

### 4.5. Statistical Analysis

Statistical analysis of protein concentrations from clinical routine analysis (CSF/serum ratios of albumin, IgG, IgA and IgM) was carried out with the Wilcoxon–Mann–Whitney test. For the determination of differentially abundant peptides, liquid chromatography coupled mass spectrometric data were statistically analyzed by analysis of variance. Only peptides present in >75% of the samples were considered for further analysis. Peptides with a *p*-value ≤0.05 were considered as significantly different. As a test for blood contamination due to BBB dysfunction, correlation analysis was performed between the concentrations of candidate peptides and CSF albumin peptides (both determined by liquid chromatography coupled mass spectrometry). The *p*-values of the Pearson correlation were adjusted by Benjamini–Hochberg correction. Peptides with a significance threshold of *p* ≤ 0.001 and a positive fold-change were considered as contamination from BBB dysfunction. For a further selection of marker peptides, Receiver operating characteristic (ROC) analyses were applied. Only peptides with an AUC above 0.7 were considered.

### 4.6. SRM Analysis—Validation

Digested CSF samples were measured using a nano-HPLC system (UltiMate 3000 RSLCnano (Thermo Fisher Scientific) in combination with a TSQ Vantage triple quadrupole mass spectrometer (Thermo Fisher Scientific). Each sample was measured twice with a different set of spiked-in heavy peptides. For each sample, a total of 1.25 µg of digested CSF proteins including 20 fmol of heavy labeled peptides was loaded at a rate of 20 µL min^−1^ for five minutes and separated on a 15-min gradient (4%–35% B, where solution A is 0.1% formic acid in water and solution B is 84% acetonitrile, 0.1% Trifluoroacetic acid), using a column of 75-µm i.d., 15-cm length, C18 and 2-µm particle size (Acclaim PepMap RSLC; Thermo Fisher Scientific). Samples were sprayed into the mass spectrometer by electrospray ionization using a spray voltage of 1.4 kV. The resolution of Q1 and Q3 was set to 0.7 u full width at half maximum. The cycle time was 1.5 s. Instrument scan mode was SRM, and three primary fragment ions per peptide were collected.

Peptides were fragmented with a threshold intensity of 100 counts by collision-induced dissociation using argon gas (1.5 mTorr pressure) at collision energies specific for each peptide. Each peptide was measured in a scheduled manner within a two-minute time interval.

Recorded mass spectrometric intensities were analyzed with Skyline 4.2.0.18305 (MacCoss Lab, University of Washington). All transitions were manually inspected and peptides showing heavy interferences not further quantitatively considered for the respective samples. Absolute amounts of CSF peptides were calculated by the ratio of light and heavy peptides, ANOVA with subsequent Tukey’s post hoc tests, marker combinations by generalized linear models and ROC curves were calculated within the R environment (R version 3.4.1, The R Foundation for Statistical Computing). For the comparison of two groups, combinations of marker peptides were used. Here, a binominal logistic regression was performed using the glm function. The measured peptide amounts were used as predictors and respective two groups as target variables. Samples for which no values were available were not considered.

### 4.7. Immunohistochemistry

Paraffin-embedded specimens of an independent series of 20 immunocompetent patients with newly diagnosed PCNSL (10 females, 10 males, age: 47–86 years, mean age: 71 years) were used for immunohistochemistry. Normal brain specimens from five surgically treated epilepsy patients were used as the control. Immunohistochemistry was performed with polyclonal rabbit anti-human apolipoprotein CII antibody (ab76452, Abcam, Cambridge, UK), polyclonal rabbit anti-human VSIG4 antibody (Nouvs Biologicals, Wiesbaden, Germany) and monoclonal rabbit anti-human GPNMB (EAD7P) XP antibody (Cell Signaling, Frankfurt, Germany) on a Leica Bond immunostainer according to the manufacturer’s instructions. Expression of the respective protein was assessed by semiquantitative evaluation according to a grading system with: −, negative; +, <10%; ++, <50%; +++, <90%; ++++, >90% of the tumor cells, respectively. The analysis was performed by two independent observers (M.D., A.B.) yielding identical results.

## 5. Conclusions

With these results, we outlined that the application of two orthogonal mass spectrometric approaches for the discovery and validation of candidate biomarkers is an unbiased alternative method in clinical diagnostics and offers an attractive route to improve the differential diagnosis of patients with PCNSL by the combination of novel peptide biomarkers as well as already established clinical parameters such as neuroimaging.

## Figures and Tables

**Table 1:** Demographic data of participating individuals. (**a**) Discovery study cohort, (**b**) validation cohort and (**c**) immunohistochemistry (IHC) cohort. For detailed diagnosis, see Table S1.

a					
Discovery Cohort					
Group	Total	Male	Female	Age (Range)	Age (Median)
PCNSL	19	9	10	49–74	60
SCNSL	9	4	5	51–63	60
Multiple sclerosis	9	4	5	36–70	51
Gliomas	10	6	4	38–76	56
Other tumors	10	5	5	41–76	62
Tumor-free control	8	4	4	52–69	60
**b**					
**Validation Cohort**					
**Group**	**Total**	**Male**	**Female**	**Age (Range)**	**Age (Median)**
PCNSL	28	10	18	38–83	65
SCNSL	3	2	1	69–79	73
Multiple sclerosis	14	3	11	18–43	27
Gliomas *	10	6	4	38–76	56
Other tumors	7	2	5	42–79	48
Tumor-free control	11	5	6	55–66	60
**c**					
**IHC Cohort**					
**Group**	**Total**	**Male**	**Female**	**Age (Range)**	**Age (Median)**
PCNSL	20	10	10	47–86	71
Tumor-free control	5	2	3	54–60	55

* identical samples as in discovery cohort.

**Table 2:** Proportion of patients with a BBB disruption and corresponding CSF/serum concentration quotients of albumin, IgG, IgA and IgM.

Group	Total Number of Patients	Patients with BBB-Disruption	Proportion	Range of CSF/Serum Ratio (mean)
**PCNSL**	19	11	58%	Alb.: 4.7–26.3 (10.9) IgG: 2.9–17.6 (6.4) IgA: 1.6–13.2 (4.3) IgM: 0.2–68.6 (6.5)
**SCNSL**	9	5	56%	Alb.: 5.6–15.2 (10.6) IgG: 0.7–9.0 (4.3) IgA: 0.3–5.1 (2.3) IgM: 0.4–1.3 (0.8)
**Gliomas**	10	6	60%	Alb.: 3.9–17.0 (9.3) IgG: 1.9–8.7 (4.2) IgA: 0.1–5.6 (2.6) IgM: 0.1–3.8 (1.1)
**Other tumors**	10	4	40%	Alb.: 3.5–85.5 (20.6) IgG: 1.2–49.4 (15.3) IgA: 0.4–31.1 (6.7) IgM: 0.1–12.3 (2.1)
**Tumor-free control**	8	1	13%	Alb.: 4.1–10.7 (6.7) IgG: 1.1–4.6 (2.8) IgA: 0.5–2.8 (1.7) IgM: 0.2–0.8 (0.4)

**Table 3:** Assignment of differentially abundant proteins to tissue origin and protein class.

Patient Group	Proteins (Total)	CNS Proteins	Pro-Portion of CNS Proteins	Membrane Proteins	Proportion of Membrane Proteins	Secreted Proteins	Proportion of Secreted Proteins
PCNSL vs. SCNSL	71	36	51%	46	65%	31	44%
PCNSL vs. Glioma	41	23	56%	23	56%	20	49%
PCNSL vs. Tumors	31	17	55%	15	48%	17	55%
PCNSL vs. multiple sclerosis	50	26	52%	30	60%	33	66%
PCNSL vs. Control	88	57	65%	57	65%	62	70%

**Table 4:** Candidate peptide biomarkers selected for the development of selected reaction monitoring (SRM) assay. PS, PCNSL vs. SCNSL; PG, PCNSL vs. glioma; PD, PCNSL vs. disease; PM, PCNSL vs. MS; PT, PCNSL vs. other tumors; PC, PCNSL vs. tumor-free control. Score: MS Amanda score (quality of identification). *p*-value: t-test between the compared groups. AUC: area under the curve calculated between the compared groups. Tissue database: UniProt tissue annotation database.

Feature	Sequence	Accession	Gene	Description	*p*-Value	AUC ^a^ (Discovery Cohort)	Diff. Analysis	Tissue	Abundance Range
909	DYESGQLYGLEK ^b^	Q659C4	LARP1B	La-related protein 1B	0.03610	0.81	PS		High
1347	TALALEVGELVK ^b^	P46108	CRK	Adapter molecule crk	0.00631	0.83	PS		Medium
2253	LEDKVK ^a^	P17275	JUNB	Transcription factor jun-B	0.02260	0.82	PS		Medium
7149	VFINLLDSYSSGNIGK ^b^	Q96M32	AK7	Adenylate kinase 7	0.01150	0.78	PG		Medium
8945	TAAQNLYEK	P02655	APOC2	Apolipoprotein C-II	0.02180	0.83	PM		Medium
10516	AGADLSLLDR ^b^	P19838	NFKB1	Nuclear factor NF-kappa-B p105 subunit	0.00279	0.86	PM		Medium
12738	WVQWFGDGK ^c^	Q9UBC3	DNMT3B	DNA (cytosine-5)-methyltransferase 3B	0.02850	0.81	PS	Brain	Medium
19446	GSDPVTIFLR	Q9Y279	VSIG4	V-set and immunoglobulin domain-containing protein 4	0.01580	0.74	PG	Brain	Medium
23266	DSFPYLEPLGAIPDVQK	Q15111	PLCL1	Inactive phospholipase C-like protein 1	0.02440	0.83	PS	Brain	Medium
23650	ADLIAYLK	P99999	CYCS	Cytochrome c	0.01900	0.85	PG	Brain	Medium
29307	DGLILTSR	Q99497	PARK7	Protein deglycase DJ-1	0.00492	0.82	PG	Brain	Medium
30944	VEFLRPSFTDGTIR ^c^	Q15223	NECTIN1	Nectin-1	0.02080	0.72	PG	Brain/Plasma	Medium
36323	SSGLVSNAPGVQIR	P04180	LCAT	Phosphatidylcholine-sterol acyltransferase	0.00055	0.91	PC	Brain/Plasma	Medium
36464	NGEEFSFLK	Q99574	SERPINI1	Neuroserpin	0.04050	0.75	PT/PC	Brain	Medium
37006	AYVPIAQVK	Q14956	GPNMB	Transmembrane glycoprotein NMB	0.00862	0.77	PG/PM	Brain/Liver	Medium
37088	EVLPAIR ^b^	O00391	QSOX1	Sulfhydryl oxidase 1	0.02580	0.73	PT	Brain/Plasma	Medium
38690	ALGFEAAESSLTK ^b^	Q8TDL5	BPIFB1	BPI fold-containing family B member 1	0.00045	0.93	PC		Medium
38843	VNHAVLAVGYGEK ^b^	P09668	CTSH	Pro-cathepsin H	0.04180	0.75	PG	Liver	Medium
39626	DQVANSAFVER ^b^	P07900	HSP90AA1	Heat shock protein HSP 90-alpha	0.03180	0.73	PG	Brain/Liver	Medium
40050	DGLIPLEIR ^c^	Q13228	SELENBP1	Selenium-binding protein 1	0.00031	0.94	PC	Brain	Medium
45513	EMDPVTQLYTMTSTLEYK ^a^	Q13740	ALCAM	CD166 antigen	0.00516	0.84	PG/PC	Brain/Plasma/Liver	Low
50614	WVGDLPNGR ^b^	Q96DR8	MUCL1	Mucin-like protein 1	0.01760	0.82	PM		Low
57938	ATYIQNYR ^c^	Q01459	CTBS	Di-N-acetylchitobiase	0.02710	0.81	PM	Brain/Liver	Medium
61240	FDAPPEAVAAK ^b^	O95969	SCGB1D2	Secretoglobin family 1D member 2	0.00203	0.83	PM	Brain	Low
62517	FRDLEEDPYLPGNPR ^b^	P22304	IDS	Iduronate 2-sulfatase	0.01920	0.78	PT	Liver	Medium
68357	AYLEVTDVIADRPPPVIR ^b^	Q9Y6N7	ROBO1	Roundabout homolog 1	0.01630	0.81	PM		Low
73987	DLAEVPASIPVNTR	Q9NT99	LRRC4B	Leucine-rich repeat-containing protein 4B	0.03940	0.71	PG	Brain	Low
89726	ISGLIYEETR ^b^	P62805	HIST1H4A	Histone H4	0.01910	0.82	PS	Brain	Medium
137254	VTDANYGELQEHKAQAYLK ^a^	Q8TAG5	VSTM2A	V-set and transmembrane domain-containing protein 2A	0.04850	0.74	PT	Brain	Low
142941	ILSGRPPLGFLNPR ^b^	O14773	TPP1	Tripeptidyl-peptidase 1	0.03580	0.82	PS	Brain/Liver	Low
177384	ESYNVQLQLPAR ^b^	Q14112	NID2	Nidogen-2	0.04400	0.75	PT	Liver	Medium
199236	SQLEAIFLR ^b^	Q8IV08	PLD3	Phospholipase D3	0.00249	0.85	PG	Brain/Liver	Low

^a^ due to oxidizable amino acids, missed cleavage sites as well as theoretical transition interference were not considered for SRM. ^b^ due to the low S/N ratio, not considered for SRM. ^c^ due to transition interferences under experimental conditions, not considered for SRM.

**Table 5:** Concentration of candidate peptide biomarkers determined in the validation cohort. The standard deviation of the biomarker peptides concentration in CSF is given in brackets.

Protein	Accession	Peptide	Control [fmol/µg]	Glioma [fmol/µg]	Multiple Sclerosis [fmol/µg]	PCNSL [fmol/µg]	SCNSL [fmol/µg]	Tumors [fmol/µg]
LRRC4B	Q9NT99	DLAEVPASIPVNTR	106.75 (92.08)	47.70 (40.22)	41.41 (40.33)	71.08 (60.99)	64.46 (98.25)	46.54 (50.42)
PARK7	Q99497	DGLILTSR	6.66 (2.76)	6.15 (2.69)	6.66 (5.49)	7.46 (3.45)	5.30 (2.24)	8.22 (8.94)
SERPINI1	Q99574	NGEEFSFLK	1.70 (0.84)	1.00 (0.56)	1.04 (0.58)	1.00 (0.70)	0.69 (0.61)	0.53 (0.36)
VSIG4	Q9Y279	GSDPVTIFLR	26.12 (19.35)	19.18 (13.56)	21.77 (27.56)	104.10 (132.02)	68.75 (103.03)	63.12 (67.76)
APOC2	P02655	TAAQNLYEK	8.44 (6.77)	5.19 (2.58)	5.96 (5.92)	16.84 (15.17)	15.48 (16.12)	18.72 (9.25)
CYCS	P99999	ADLIAYLK	5.10 (3.19)	3.12 (1.60)	3.62 (2.92)	3.46 (2.22)	2.04 (0.97)	7.29 (12.64)
GPNMB	Q14956	AYVPIAQVK	0.33 (0.20)	0.23 (0.06)	0.24 (0.12)	0.55 (0.41)	0.25 (0.07)	0.41 (0.30)
LCAT	P04180	SSGLVSNAPGVQIR	3.55 (2.15)	2.29 (1.13)	2.87 (1.94)	2.95 (1.58)	1.82 (0.25)	2.17 (1.12)

**Table 6:** Results from the SRM analysis of the independent validation cohort. The values in bold letters assign significant abundant changes.

Protein	Accession	Peptide	ANOVA *p*-Value	PCNSL vs. Control	PCNSL vs. Glioma	PCNSL vs. Multiple Sclerosis	PCNSL vs. SCNSL	PCNSL vs. Tumors
LRRC4B	Q9NT99	DLAEVPASIPVNTR	0.1603	0.8443	0.9988	0.5417	0.8747	0.8312
PARK7	Q99497	DGLILTSR	0.6558	0.9997	0.9866	0.5067	0.9845	0.9983
SERPINI1	Q99574	NGEEFSFLK	**0.0298**	0.1350	0.9982	0.9976	0.9386	0.6346
VSIG4	Q9Y279	GSDPVTIFLR	**0.0009**	0.1540	**0.0339**	**0.0006**	0.8844	0.9495
APOC2	P02655	TAAQNLYEK	**0.0001**	0.2592	**0.0124**	**0.0008**	0.9991	0.9924
CYCS	P99999	ADLIAYLK	0.8286	0.9319	1.0000	0.9995	0.9667	1.0000
GPNMB	Q14956	AYVPIAQVK	**0.0027**	0.2994	**0.0191**	**0.0049**	0.4793	0.7925
LCAT	P04180	SSGLVSNAPGVQIR	0.7455	0.9989	0.9902	0.8298	0.9889	0.9743

**Table 7:** Patients data and protein expression in PCNSL tissue (#1–20) and in normal brain (#21–25), respectively. Protein expression in tumor cells: −: Tumor cells negative; +: <10% of tumor cells positive; ++: 10–40% of tumor cells positive; +++: >40–80% of tumor cells positive; ++++: >80% of tumor cells positive.

Case	Sex	Age	Apolipoprotein CII	GPNMB	VSIG4
1	f	77	−	−	−
2	m	79	−	−	−
3	f	71	−	++	−
4	m	66	−	−	−
5	m	86	−	++	−
6	f	66	++	++	−
7	f	70	−	++	−
8	f	80	−	−	−
9	f	76	−	−	−
10	f	69	−	+	−
11	m	60	−	−	−
12	m	47	−	−	−
13	m	79	−	−	−
14	m	69	−	−	−
15	f	76	−	−	−
16	m	78	−	−	−
17	f	75	−	+++	−
18	f	60	−	+	−
19	m	70	+	−	−
20	m	70	−	−	−
21	f	54	neurons +	neurons +, astrocytes +, oligodendrocytes weakly +	astrocytes +
22	f	55	neurons +	astrocytes + microglial cells +	astrocytes +
23	f	60	neurons +	astrocytes + microglial cells +	astrocytes +
24	m	54	neurons +	microglial cells +	astrocytes +
25	m	58	neurons +	microglial cells +	astrocytes +

## Supplementary Materials

The following are available online at https://www.mdpi.com/2072-6694/12/7/1732/s1, Figure S1: Results of ROC analysis. Colors indicate different peptides originating from the respective comparison (Table 4). (a) PCNSL vs. SCNSL (b) PCNSL vs. glioma (c) PCNSL vs. “other tumors” (d) PCNSL vs. multiple sclerosis (e) PCNSL vs. non-tumor controls (f) Venn diagram of candidate biomarkers. Table S1A: Demographic data of participating individuals of discovery study cohort, Table S1B: Demographic data of participating individuals of validation cohort, Table S2: Results of discovery study, Table S3: Results of tissue annotation from UniProt tissue annotation database.
