# Supplementary figures and images for: A Multiplex Assay for the Stratification of Patients with Primary Central Nervous System Lymphoma Using Targeted Mass Spectrometry
Source: Cancers (Basel). 2020 Jun 29;12(7):1732. doi: 10.3390/cancers12071732 (PMC7407338; doi:10.3390/cancers12071732)

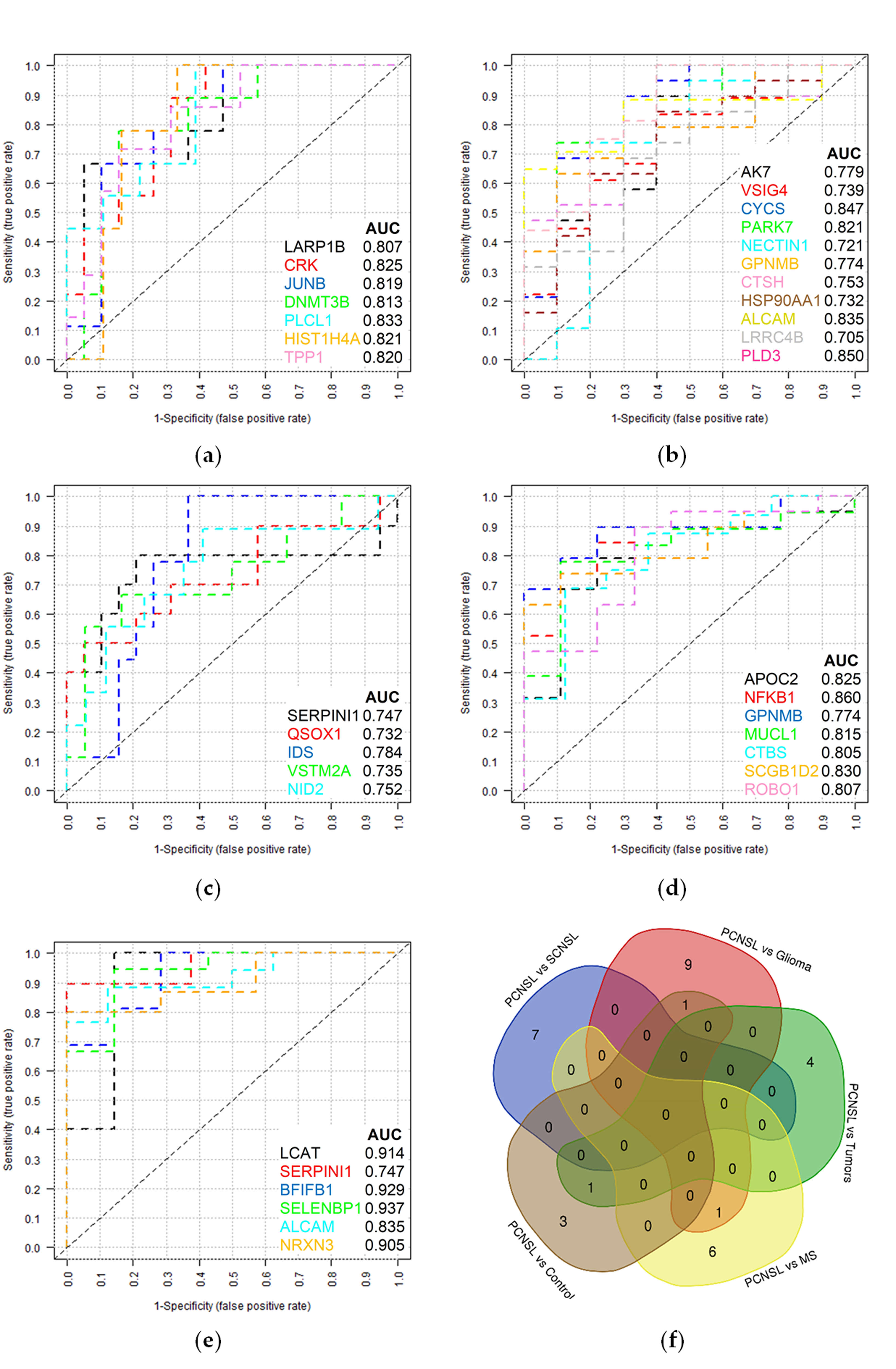

Supplement: Supplementary file 1 [file cancers-12-01732-s001.zip › Supplement/SFigure1.jpg]
